# Supplementary material for: Rare sugar l-sorbose exerts antitumor activity by impairing glucose metabolism
Source: Commun Biol. 2023 Mar 11;6:259. doi: 10.1038/s42003-023-04638-z (PMC10008635; doi:10.1038/s42003-023-04638-z)
Supplement: Supplementary file 2 — supplementary Figures [file 42003_2023_4638_MOESM2_ESM.pdf]

Supplementary Fig. 1

a

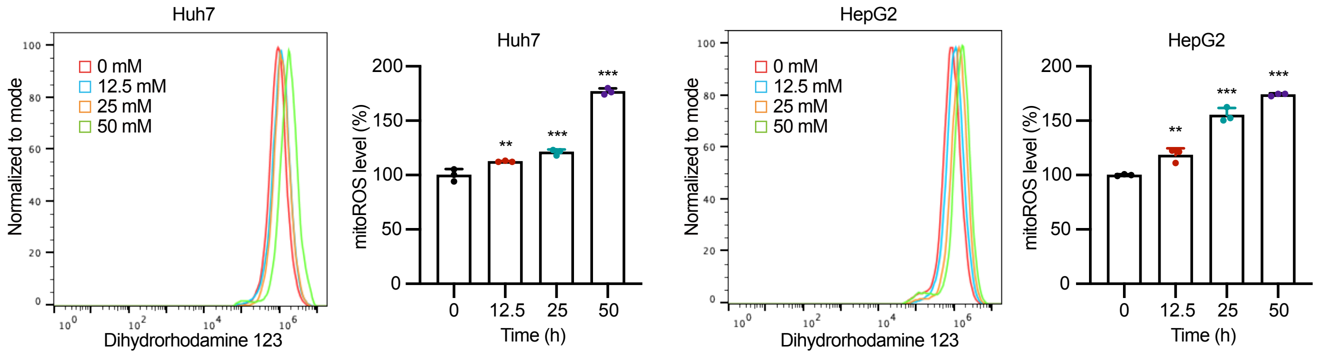

b

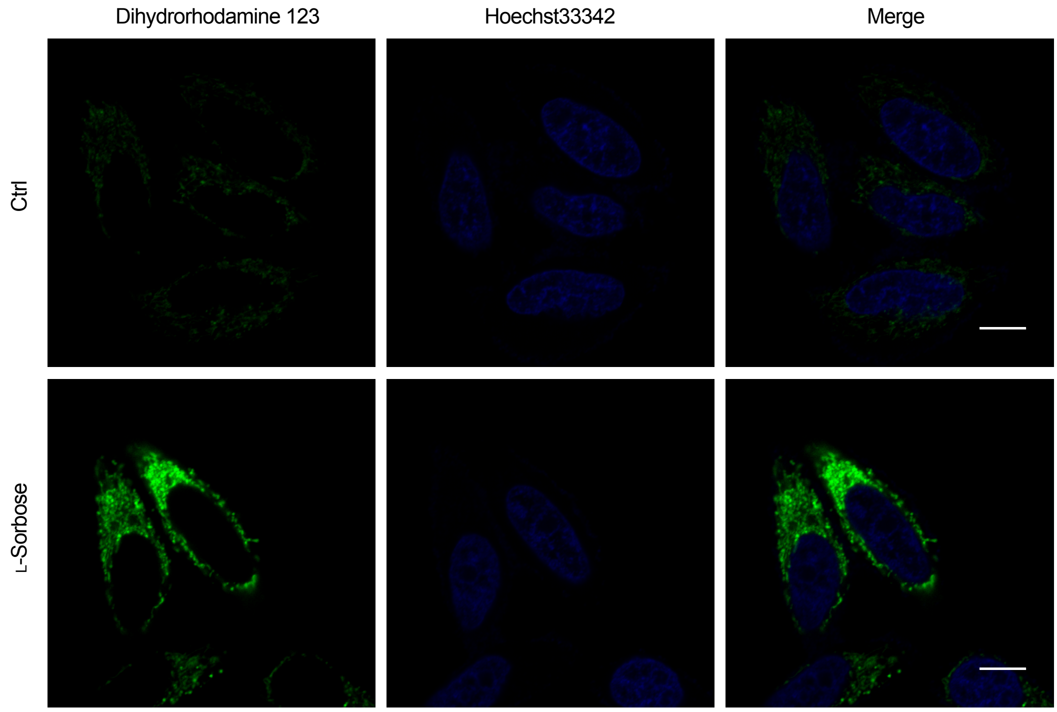

Supplementary Fig. 1 L-Sorbose enhances mitochondrial ROS accumulation in cancer cells. a, The mitochondrial ROS level was detected by Dihydrorhodamine 123 after 0, 12.5, 25 or 50 mM L-sorbose treatment for 24 h. Left panels: Representative histograms. Right panels: Quantification of the MFI values. The data were normalized to the cells without L-sorbose treatment group (100%). n = 3. **b**, Representative images of Huh7 cells stained with Dihydrorhodamine 123 (green) and Hoechst33342 (blue) after 0 or 25 mM L-sorbose treatment for 6 h. Scale bar, 10 μm. Data are presented as the mean ± s.d. and were analyzed by unpaired two-tailed Student's t test. $^{**}P < 0.01$, $^{***}P < 0.001$.

Supplementary Fig. 2

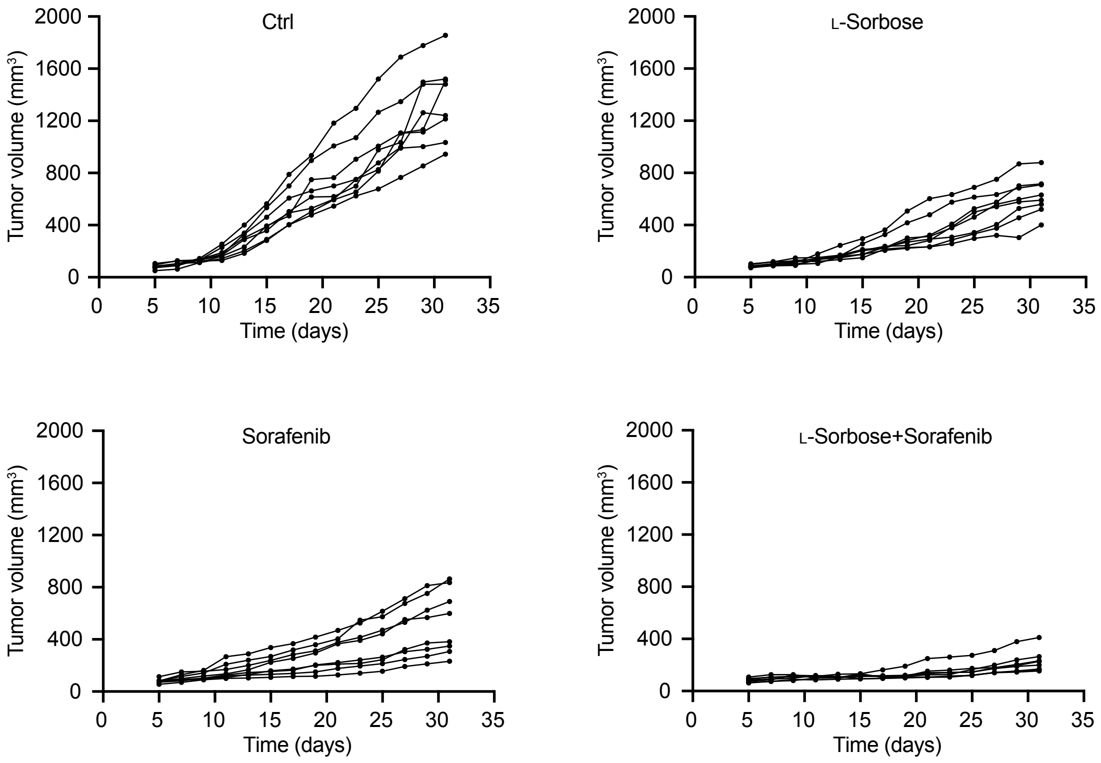

Supplementary Fig. 2 L-Sorbose enhances the anticancer activity of sorafenib. BALB/c nude mice were injected with Huh7 cells subcutaneously and received either normal drinking water or 20% L-sorbose by oral gavage every day from the fifth day after tumor transplantation. Sorafenib was intragastrically administered to mice at a dose of 50 mg/kg every day from the day eleventh. The tumor volume of each mouse was measured.

Supplementary Fig. 3

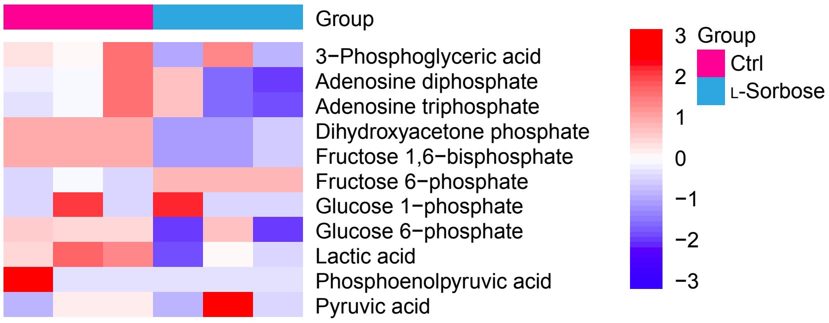

Supplementary Fig. 3 L-Sorbose treatment influences the glucose metabolic flux of Huh7 cells. Extraction of intracellular metabolites and measurement of the abundence of uniformly labeled metabolites of Huh7 cells after 12 h incubation in glucose-free DMEM complete medium in the presence of $^{13}C_6$-D-glucose. n = 3.

Supplementary Fig. 4

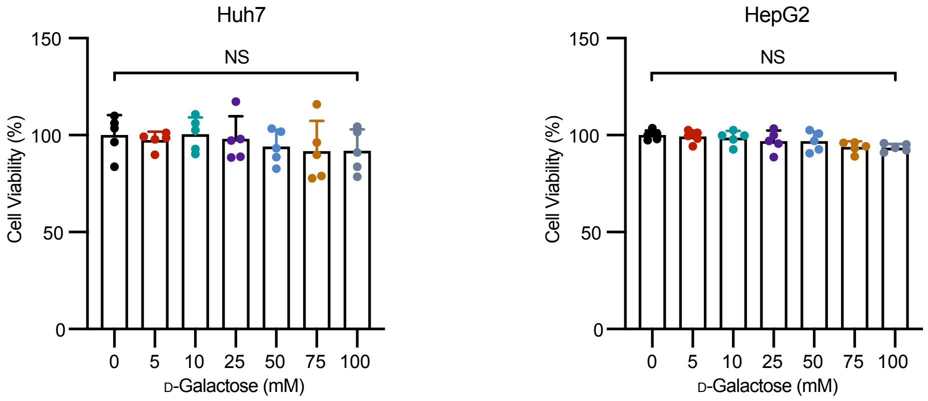

Supplementary Fig. 4 Galactose does not influence cell viability. Viabilities of cells treated with the indicated concentrations of D-galactose for 24 h. n = 5. Data are presented as the mean ± s.d. and were analyzed by one-way ANOVA with Dunnett's test. NS, not significant.

Supplementary Fig. 5

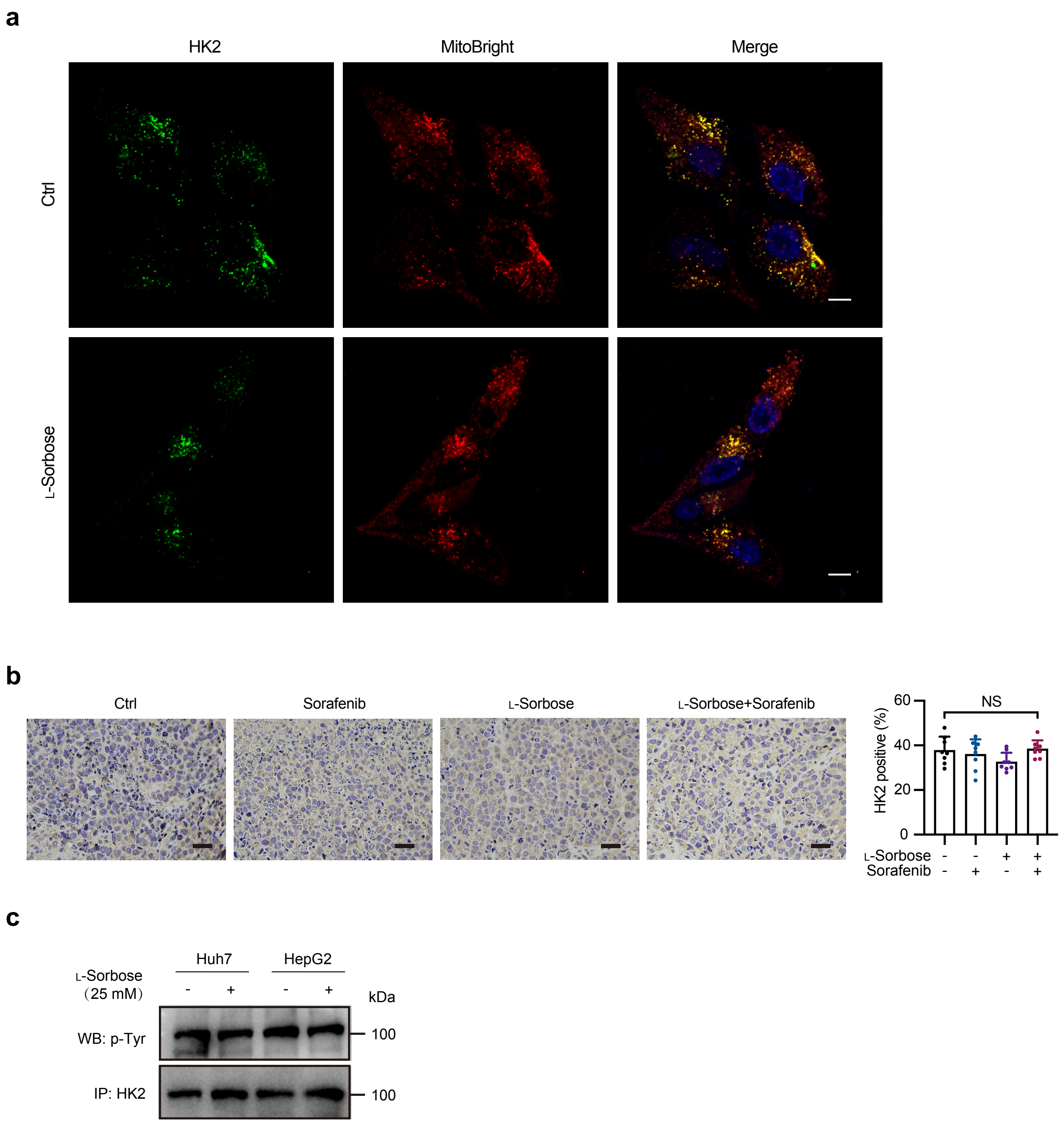

Supplementary Fig. 5 HK2 localization and expression were not altered by L-sorbose treatment. a, Representative images of Huh7 cells stained with anti-HK2 antibody (green), mitochondrial marker MitoBright (red) and Hoechst33342 (green) after 0 or 25 mM L-sorbose treatment for 24 h. Scale bar, 10 μm. **b**, Left panels: Representative images of HK2 IHC staining of mice tumor tissues. Right panels: Quantification of HK2 positive percentage. Scale bar, 50 μm. n = 8. **c**, Cells were treated with L-sorbose, followed by IP for HK2 and western blot (WB) with a pan anti-phospho-tyrosine antibody (Anti-p-Tyr) to detect the HK2 phosphorylation. Data are presented as the mean ± s.d. and were analyzed by unpaired two-tailed Student's t test. NS, not significant.

Supplementary Fig. 6

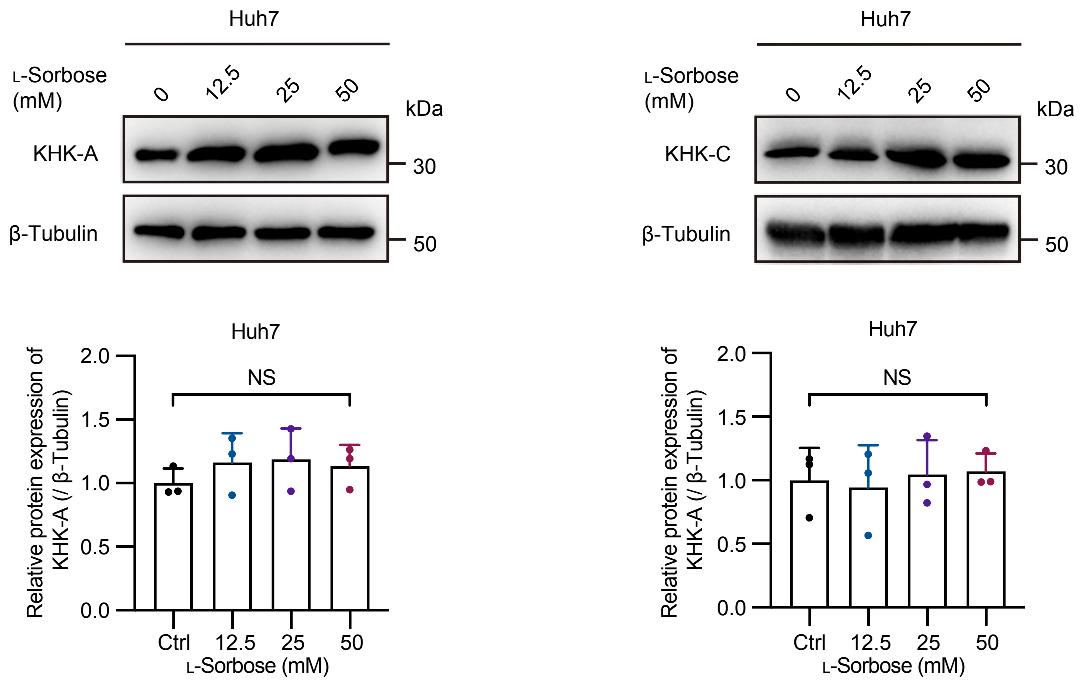

Supplementary Fig. 6 L-Sorbose has no influence on the expression of KHK-A and KHK-C in a D-galactose environment without glucose. Top panels: Western blot of KHK-A and KHK-C levels in cells cultured with glucose-free DMEM supplemented with D-galactose after being treated with L-sorbose. Bottom panels: Relative intensities of KHK-A and KHK-C. The intensity of KHK-A and KHK-C normalized to β-tubulin detected in cells without L-sorbose treatment were defined as 1. n = 3. Data are presented as the mean ± s.d. and were analyzed by unpaired two-tailed Student's t test. NS, not significant.

Supplementary Fig. 7

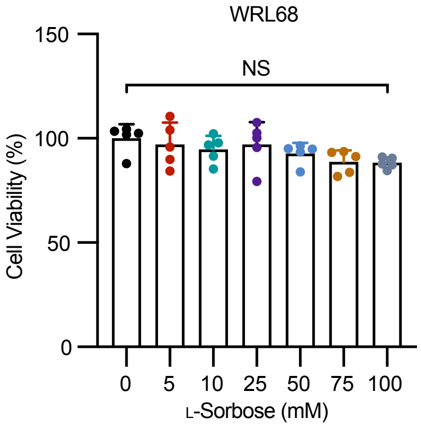

Supplementary Fig. 7 L-Sorbose has no influence on cell viability in WRL68 cells. Viabilities of cells treated with L-sorbose for 24 h. n = 5. Data are presented as the mean ± s.d. and were analyzed by one-way ANOVA with Dunnett's test.

Supplementary Fig. 8

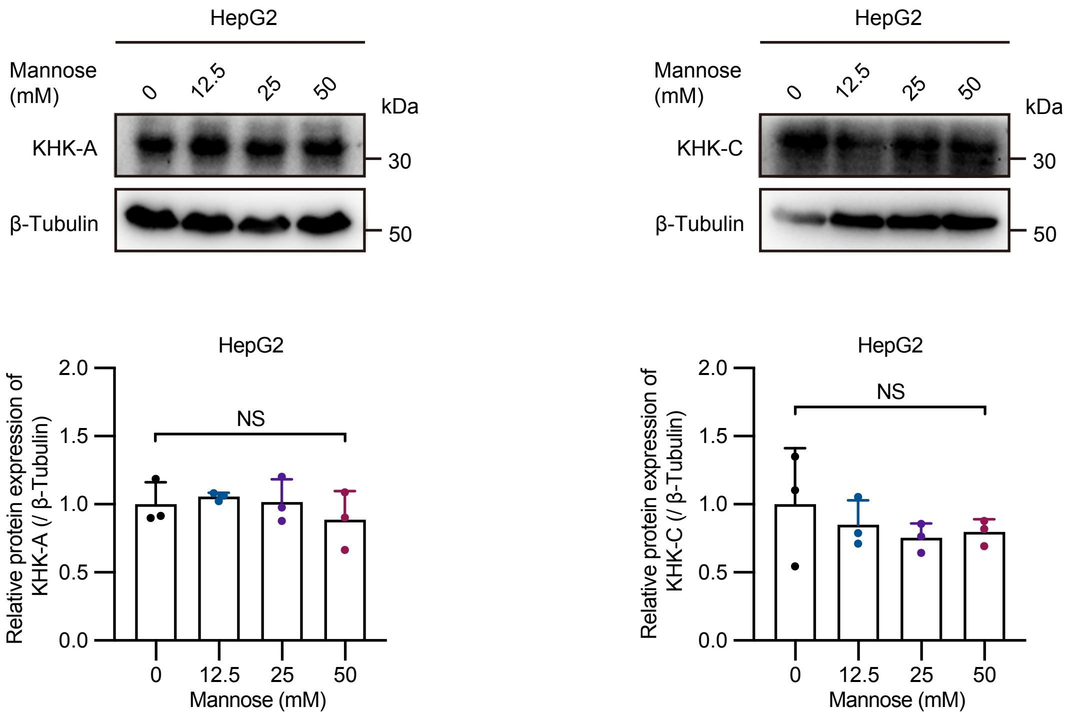

Supplementary Fig. 8 Mannose does not affect KHK levels. Top panels: Western blot of KHK-A and KHK-C in HepG2 cells after treatment with 0, 12.5, 25 or 50 mM mannose for 24 h. Bottom panels: Relative intensities of KHK-A and KHK-C. The intensity of KHK-A and KHK-C normalized to β-tubulin detected in cells without mannose treatment were defined as 1. n = 3. Data are presented as the mean ± s.d. and were analyzed by unpaired two-tailed Student's t test. NS, not significant.

Supplementary Fig. 9 Unprocessed western blots

Fig. 1e

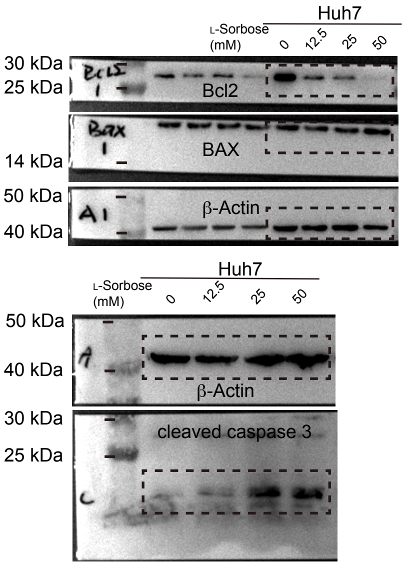

All samples were loaded with the same amount.

Fig. 4d

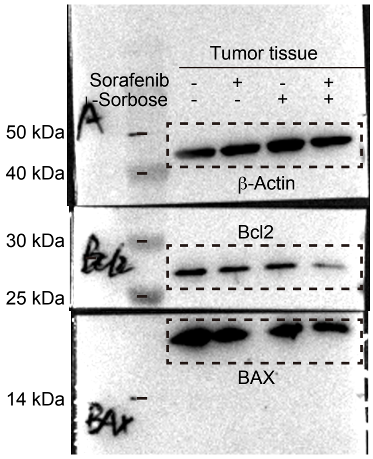

Fig. 5c

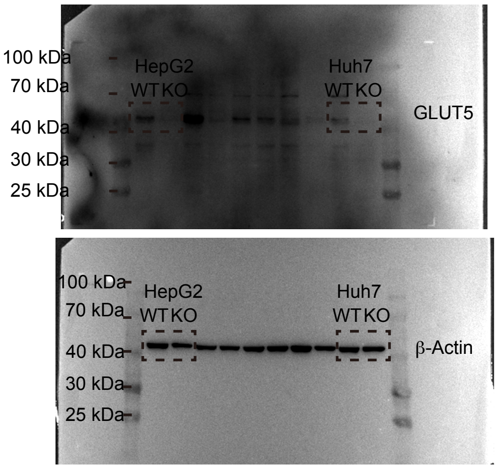

All samples were loaded with the same amount.

Fig. 5f

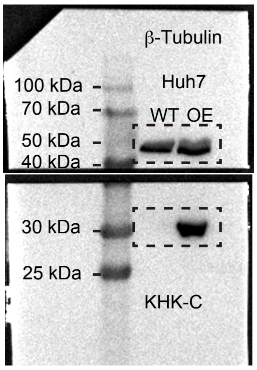

Fig. 6d

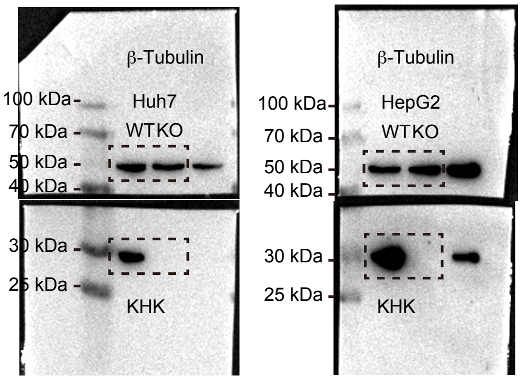

Fig. 6e

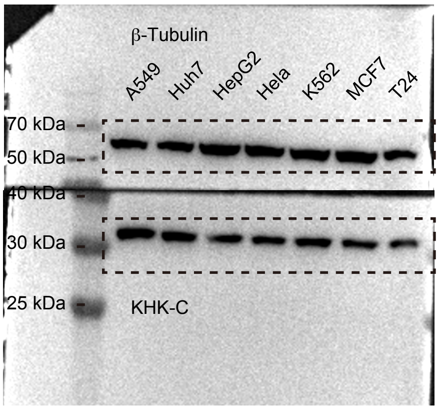

Fig. 6j

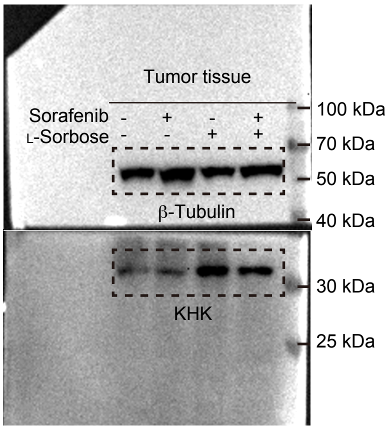

Fig. 7e

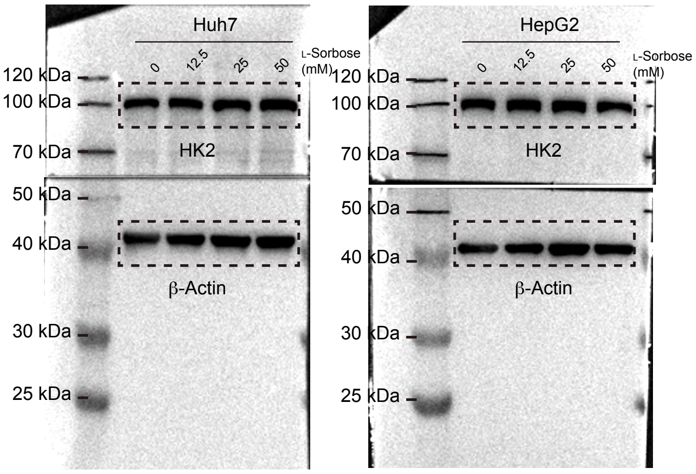

Fig. 7f

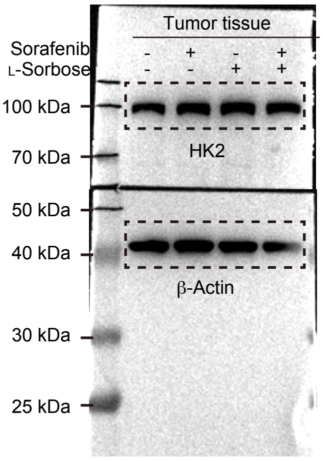

Supplementary Fig. 10 Unprocessed western blots

Fig. 8b

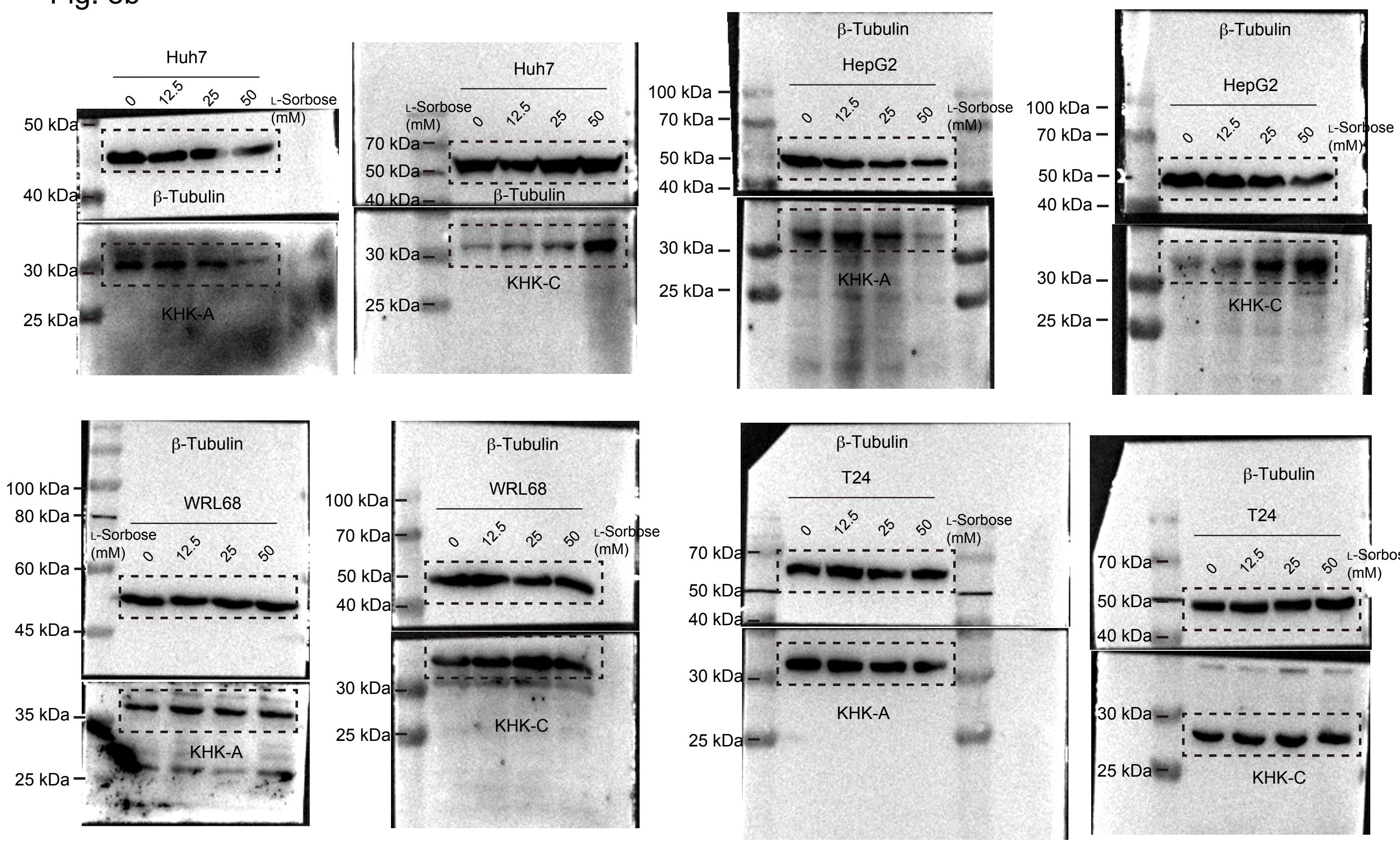

Fig. 8c

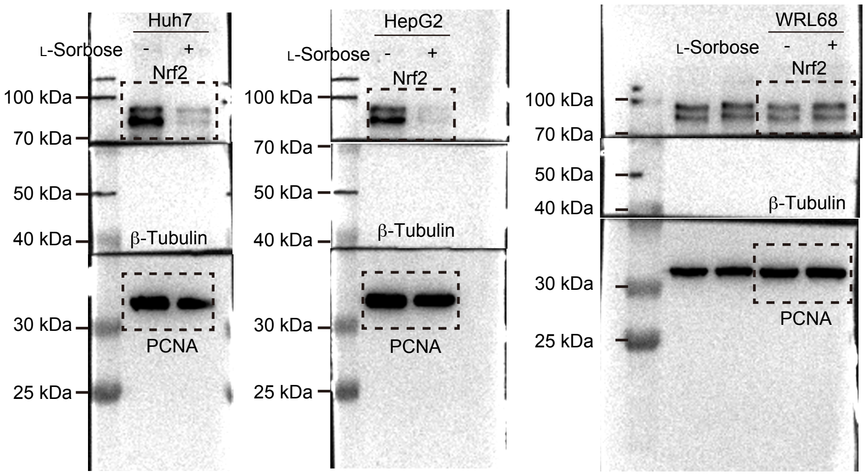

Supplementary Fig. 5c

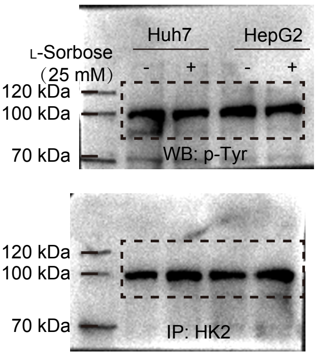

Supplementary Fig. 6

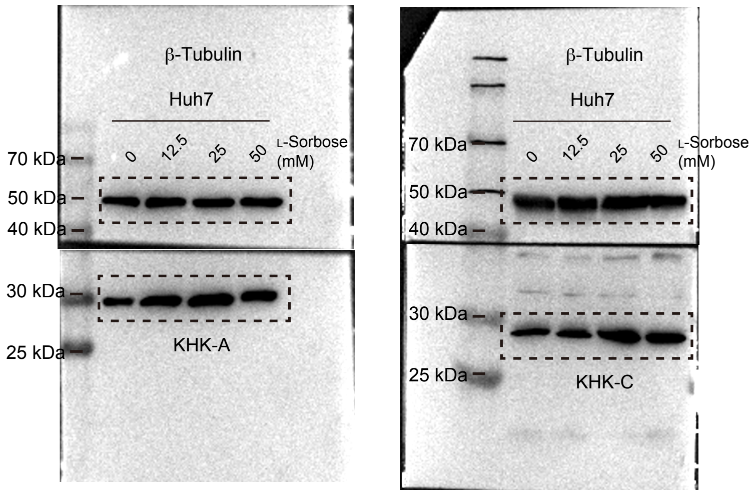

Supplementary Fig. 8

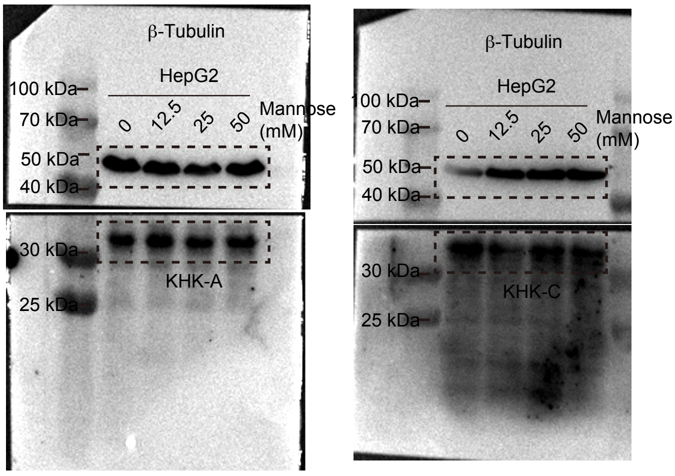

Supplementary Table 1 Plasmids used in this study

Plasmids	Parental plasmids	Description	Cloning sites
pLVX-*KHK-C*-puro	pLVX-puro	pLVX-pure harboring *KHK* gene	*Eco*RI/*Not*I
pET28a-2His-*KHK*	pET28a-2His	pET28a-2His harboring *KHK* gene	*Bam*HI/*Hind*III
pLVX-CRISPR-v2-*SLC2A5*-puro	pLVX-CRISPR-v2-puro	pLVX-CRISPR-v2-puro bearing S*LC2A5* guide RNAs	*Bsm*BI
pLVX-CRISPR-v2-*KHK*-puro	pLVX-CRISPR-v2-puro	pLVX-CRISPR-v2-puro bearing *KHK* guide RNAs	*Bsm*BI
psPAX.2	psPAX.2	For lentivirus transfection	--
pMD2.G	pMD2.G	For lentivirus transfection	--

Supplementary Table 2 Guide RNA sequences

Gene name	Direction	Sequence of guide RNA
SLC2A5	Forward	5’-GATGCGCTCGCTGCGCTGGC-3’
	Reverse	5’-GCCAGCGCAGCGAGCGCATC-3’
KHK	Forward	5’-GGTGCTGGACGTCATCAGCC-3’
	Reverse	5’-GGCTGATGACGTCCAGCACC-3’

Supplementary Table 3 Primers for quantitative real-time PCR

Gene name	Direction	Sequence
HO-1	Forward	GGTCAGGTGTCCAGAGAAGG
	Reverse	CTTCCAGGGCCGTGTAGATA
NQO1	Forward	ATCCTGCCGAGTCTGTTCTG
	Reverse	AGGGACTCCAACCACTGC
GCLC	Forward	GTGGACGAGTGCAGCAAG
	Reverse	GTCCAGGAAATACCCCTTCC
PGD	Forward	AAAGATCCGGGACAGTGCT
	Reverse	CACCGAGCAAAGACAGCTT
KHK-A	Forward	TATTCTGTGGACCTACGCTA
	Reverse	CATAGTATAGGATGGTGCGG
KHK-C	Forward	CATGTTGCTGACTTCCTGG
	Reverse	TTGGAGTTGTTGATGATGCA
GAPDH	Forward	GAGTCAACGGATTTGGTCGT
	Reverse	TTGATTTTGGAGGGATCTCG
β-Actin	Forward	CATCACCATCTTCCAGGAG
	Reverse	AGGCTGTTGTCATACTTCTC
