# Supplementary material for: Rare sugar l-sorbose exerts antitumor activity by impairing glucose metabolism
Source: Commun Biol. 2023 Mar 11;6:259. doi: 10.1038/s42003-023-04638-z (PMC10008635; doi:10.1038/s42003-023-04638-z)
Supplement: Supplementary file 4 — reporting summary [file 42003_2023_4638_MOESM4_ESM.pdf]

nature portfolio

Corresponding author(s): Xiao-Dong Gao, Hideki Nakanishi, Zijie Li

Last updated by author(s): Feb. 14, 2023

Reporting Summary

Nature Portfolio wishes to improve the reproducibility of the work that we publish. This form provides structure for consistency and transparency in reporting. For further information on Nature Portfolio policies, see our Editorial Policies and the Editorial Policy Checklist.

Statistics

For all statistical analyses, confirm that the following items are present in the figure legend, table legend, main text, or Methods section.

n/a	Confirmed	
☐	☒	The exact sample size (*n*) for each experimental group/condition, given as a discrete number and unit of measurement
☐	☒	A statement on whether measurements were taken from distinct samples or whether the same sample was measured repeatedly
☐	☒	The statistical test(s) used AND whether they are one- or two-sided<br>*Only common tests should be described solely by name; describe more complex techniques in the Methods section.*
☒	☐	A description of all covariates tested
☒	☐	A description of any assumptions or corrections, such as tests of normality and adjustment for multiple comparisons
☐	☒	A full description of the statistical parameters including central tendency (e.g. means) or other basic estimates (e.g. regression coefficient) AND variation (e.g. standard deviation) or associated estimates of uncertainty (e.g. confidence intervals)
☐	☒	For null hypothesis testing, the test statistic (e.g. F, t, r) with confidence intervals, effect sizes, degrees of freedom and P value noted<br>*Give P values as exact values whenever suitable.*
☒	☐	For Bayesian analysis, information on the choice of priors and Markov chain Monte Carlo settings
☒	☐	For hierarchical and complex designs, identification of the appropriate level for tests and full reporting of outcomes
☒	☐	Estimates of effect sizes (e.g. Cohen's *d*, Pearson's *r*), indicating how they were calculated

Our web collection on statistics for biologists contains articles on many of the points above.

Software and code

Policy information about availability of computer code

Data collection	Thermo scientific Dionex ICS-6000 HPIC system, WATERS ACQUITY UPLC System, Tanon-5200Multi, BIO-RAD microplate reader, BD Accuri C6 FACSCalibur flow cytometer
Data analysis	AB SCIEX Analyst workstation software (1.6.3 AB SCIEX), MassLynx V4.2 software, GraphPad Prism 8 (v 8.2.0), FlowJo (v 10.4.0), Image J (v 1.51)

For manuscripts utilizing custom algorithms or software that are central to the research but not yet described in published literature, software must be made available to editors and reviewers. We strongly encourage code deposition in a community repository (e.g. GitHub). See the Nature Portfolio guidelines for submitting code & software for further information.

Data

Policy information about availability of data

All manuscripts must include a data availability statement. This statement should provide the following information, where applicable:

- Accession codes, unique identifiers, or web links for publicly available datasets
- A description of any restrictions on data availability
- For clinical datasets or third party data, please ensure that the statement adheres to our policy

Data and materials supporting the findings of this study are available within the article and its supplementary information files.

Field-specific reporting

Please select the one below that is the best fit for your research. If you are not sure, read the appropriate sections before making your selection.

☒ Life sciences ☐ Behavioural & social sciences ☐ Ecological, evolutionary & environmental sciences

For a reference copy of the document with all sections, see nature.com/documents/nr-reporting-summary-flat.pdf

Life sciences study design

All studies must disclose on these points even when the disclosure is negative.

Sample size	Sample size were chosen based on the previous experience and publications. All experiments were verified at least three independent experiments. Please refer to the Figure Legends and Methods sections for further detils.
Data exclusions	All data were included, without specific exclusion.
Replication	All experiments were reproducible. Specific number of repetitions is provided in the manuscript.
Randomization	We used age and gender matched animals for the experiment. Animals were randomized assigned to each group.
Blinding	Analyses of in vivo samples were binded and analyzed by two researchers independently.

Reporting for specific materials, systems and methods

We require information from authors about some types of materials, experimental systems and methods used in many studies. Here, indicate whether each material, system or method listed is relevant to your study. If you are not sure if a list item applies to your research, read the appropriate section before selecting a response.

Materials & experimental systems

n/a	Involved in the study	
☐	☒	Antibodies
☐	☒	Eukaryotic cell lines
☒	☐	Palaeontology and archaeology
☐	☒	Animals and other organisms
☒	☐	Human research participants
☒	☐	Clinical data
☒	☐	Dual use research of concern

Methods

n/a	Involved in the study	
☒	☐	ChIP-seq
☐	☒	Flow cytometry
☒	☐	MRI-based neuroimaging

Antibodies

Antibodies used	β-Actin (Proteintech, 23660-1-AP, 1:1000), BAX (CST, 2772T, 1:1000), Bcl-2 (CST, 3498T, 1:1000), β-Tubulin (Proteintech, 10068-1-AP, 1:1000), PCNA (BBI, D220014, 1:1000), Nrf2 (Abcam, ab137550, 1:1000), HK2 (Proteintech, 66974-1-Ig, 1:5000), GLUT5 (Santa Cruz, sc-271005, 1:1000), p-Tyr (CST, 9411, 1:1000), KHK (Santa Cruz, sc-377411, 1:1000), KHK-A (SAB, 21709, 1:500), KHK-C (SAB, 21708, 1:500)
Validation	All antibodies are commercially available and validated for indicated applications and passed QC controls.

Eukaryotic cell lines

Policy information about cell lines

Cell line source(s)	The cell line WRL68 was purchased from Mingzhou Biotechnology. Other cell lines were obtained from the Cell Bank of the Type Culture Collection of Chinese Academy of Sciences.
Authentication	Cell lines used in this study were authentication by STR profiling.
Mycoplasma contamination	Cells have been checked for mycoplasma every two weeks and all cell lines used in this study were mycoplasma negative.
Commonly misidentified lines (See ICLAC register)	The cell line WRL68 is part of the list but have been authentication via STR profiling.

Animals and other organisms

Policy information about studies involving animals; ARRIVE guidelines recommended for reporting animal research

Laboratory animals	Male BALB/c nude mice aged 5 weeks were purchased. Mice were 6 weeks old when performing the experiments.
Wild animals	This study does not include any wild animal.
Field-collected samples	This study does not include samples collected from the field.
Ethics oversight	All procedures were conducted in compliance with all the relevant ethical regulations and were approved by the Jiangnan University Animal Welfare and Ethical Review Body.

Note that full information on the approval of the study protocol must also be provided in the manuscript.

Flow Cytometry

Plots

Confirm that:

- [ ] The axis labels state the marker and fluorochrome used (e.g. CD4-FITC).
- [x] The axis scales are clearly visible. Include numbers along axes only for bottom left plot of group (a 'group' is an analysis of identical markers).
- [x] All plots are contour plots with outliers or pseudocolor plots.
- [x] A numerical value for number of cells or percentage (with statistics) is provided.

Methodology

Sample preparation	Apoptosis: Cells were seeded in 6-well culture plates. After incubation with different treatment, the cells were washed twice with Annexin V binding buffer and double-stained with Annexin V and PI. Samples were then sent to flow device for analysis.<br>Measurement of ROS: Cells were cultured in 6-well culture plates. After being treated with sorbose, the cells were gently washed with D-PBS followed by incubation with DCFH-DA or Dihydrorhodamine 123.<br>Mitochondrial volume: Cells were cultured in 6-well plates. After being treated with sorbose, the cells were gently washed with D-PBS followed by incubation with MitoBright. Then the cells were washed twice with D-PBS.
Instrument	BD Accuri C6
Software	FlowJo 10.4.0 software
Cell population abundance	Total 3-4 million cells were used for sorting per sample.
Gating strategy	FSC-A versus SSC-A was applied to the gate for lymphocytes. Cells were gated on single cells using FSC-A versus FSC-H and then used for FL-2-A (PI) and FL-1-A (Annexin V-FITC) to make subsequent gates for apoptosis analysis.

- [ ] Tick this box to confirm that a figure exemplifying the gating strategy is provided in the Supplementary Information.
